# Motion in the depth direction appears faster when the target is closer to the observer

**DOI:** 10.1007/s00426-024-02040-w

**Published:** 2024-11-20

**Authors:** Yusei Yoshimura, Tomohiro Kizuka, Seiji Ono

**Affiliations:** Faculty of Health and Sport Sciences, University of Tsukuba, 1-1-1, Tennodai, Tsukuba, Ibaraki 305-8574 Japan

## Abstract

The target velocity at the retina and the initial phase of target motion are known to affect the perceived velocity of a target in planar motion. For depth motion, however, the role of this information in velocity perception remains unclear. Therefore, the purpose of this study was to reveal the role of the angular velocity derived from the vergence angle and the initial phase of target motion on the perceived velocity for depth motion. We devised two experimental tasks with five stimuli and used a two-alternative forced-choice paradigm to investigate velocity perception. In the tasks, a target moving toward or away from the observer was used. The five stimuli in each task moved between 40 and 240 cm (standard stimulus), 20 and 240 cm, 20 and 220 cm, 40 and 260 cm, and 60 and 260 cm from the participants. In the comparison of the standard stimulus with other stimuli, the stimuli approaching or receding from a distance of 20 cm were perceived as faster than the standard stimulus approaching or receding from a distance of 40 cm. We also showed that the stimuli that receded starting from a distance of 60 cm were perceived as moving slower than the standard stimulus. Our results suggest that larger changes in angular velocity affect velocity perception for depth motion; thus, observers perceive the target velocity as faster when the target is closer to the observer.

**Supplementary Information:**

The online version contains supplementary material available at 10.1007/s00426-024-02040-w.

## Introduction

It is important to perceive the velocity of a target in depth motion to prevent collisions with the target or to intercept it. The perception of the velocity of a visual target involves motion signals from the retina (Dichgans et al., 1975; Nefs & Harris, 2007). For depth motion, however, the motion of the target perceived at the retina is not the same as the motion that occurs in the real world. For example, when a visual target approaches an observer at a constant speed in the real world, the target on the retina moves with exponential acceleration. This is because the vergence angle increases exponentially as the target moves closer. Here, we defined the vergence angle as the angle between both eyes and a visual target; according to this definition, the angles at both eyes would have to converge to fixate on a target. It is still uncertain whether the target motion (angular velocity) is influenced by the vergence angle in the perception of the velocity of a target in the case of depth motion. In our previous study, we investigated the effects of the target velocity in the real world and the angular velocity on the perception of the velocity of an approaching target (Yoshimura et al., 2023). In this previous study, we used a target that moved at a constant speed in the real world and compared the velocity perception among three conditions: the target approached from 80 to 60 cm away (far condition), 60 to 40 cm away (middle condition), or 40 to 20 cm away (near condition) from the participants. The results indicated that there was no difference in the perceived velocity between the far and middle conditions and that the velocity was perceived as faster in the near condition than in the far and middle conditions. These results suggest that velocity perception depends on the target position. When a target approaches an observer, the perceived velocity depends on larger changes in the angular velocity, whereas when the target position is far from the observer, small changes in the angular velocity do not necessarily impact the perceived velocity of the target. It is also possible that the perceived velocity increases as the target approaches the observer due to the threat from the approaching target to the observer rather than changes in the angular velocity. Previous studies have demonstrated that as a threatening visual stimulus approaches an observer, the time-to-contact estimation is reduced (Brendel et al., 2012, 2014; DeLucia et al., 2014; Vagnoni et al., 2012). Thus, as the target approaches the observer, the velocity might be perceived to be faster regardless of the change in the angular velocity. Therefore, the role of the angular velocity in velocity perception for depth motion is still unclear.

Another issue that needs to be addressed is the importance of visual information in the initial phase of depth motion. Previous studies on velocity and duration perception in planar motion have demonstrated the importance of the initial phase of target motion. For example, Goettker and colleagues showed that velocity perception in planar motion depends on the pattern of eye movements in the initial phase (Goettker et al., 2018, 2019). Sasaki and colleagues reported that the perceived duration of a moving stimulus depends on the speed in the early part of the stimulus motion (Sasaki et al., 2013). Thus, if the role of the initial phase of target motion in velocity perception in depth motion is similar to that in planar motion, visual information on the initial phase of depth motion could be important for velocity perception. However, if the angular velocity has a strong effect on velocity perception with depth motion, the initial phase information may not necessarily be important for velocity perception. This is because the angular velocity information constantly changes during depth motion, even when the target moves at a constant speed in the real world. Therefore, the importance of visual information in the initial phase of depth motion remains uncertain.

The purpose of this study was to reveal the role of angular velocity and the initial phase of the target motion in velocity perception during depth motion. To achieve this purpose, we devised two experimental tasks with five types of stimuli. In one task (Experiment 1), we used a target moving from a distance toward an observer. In the other task (Experiment 2), a target moving away from the observer was used. The five stimuli used in this experiment had different initial and/or final target positions, which means that the rate of change in the angular velocity differed in the initial and/or final phases of the target motion (detailed in Methods).

## Experiment 1

### Methods

#### Participants

Eleven participants (6 men and 5 women; 24.8 ± 2.3 years old) volunteered to take part in this experiment. They reported having normal or corrected-to-normal vision and no known neurological or oculomotor disorders. Written informed consent was obtained from all participants before their participation. This study was conducted in accordance with the 2013 Declaration of Helsinki, and all experimental protocols were approved by the Research Ethics Committee at the Faculty of Health and Sport Sciences, University of Tsukuba.

#### Stimulus design

**Fig. 1:**
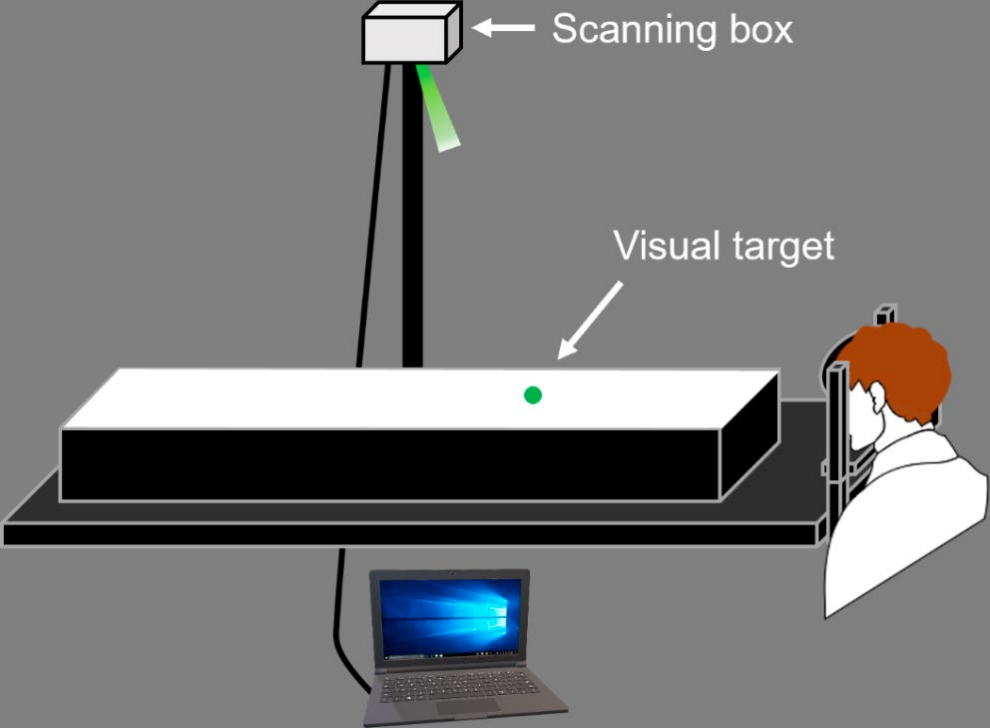
The conditions for the experimental task. A visual target moved in the depth direction

The stimulus was presented via a scanning box (Novanta Japan Cor., TY) with a galvanometer mirror and a green laser (Fig. 1). The laser in the box was irradiated to the mirror in the box, which reflected the laser onto a horizontal screen placed 2 m below the box. The green laser projected on the screen was circular with a diameter of approximately 10 mm. The stimulus was controlled via the Scan Master Designer and Scan Master Controller (Novanta Japan Cor., TY).

**Fig. 2:**
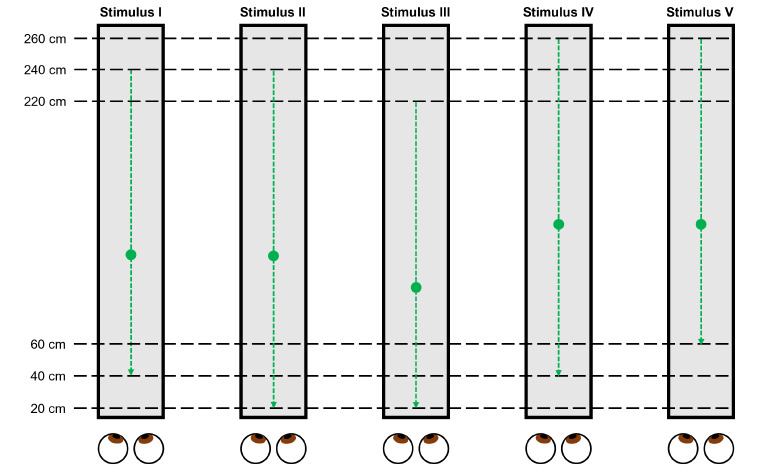
Moving distances of the visual stimuli in Experiment 1. Stimuli I, II, III, IV and V are shown. The black dashed lines indicate the distances from a participant in the depth direction. The green circles indicate the visual targets. The green dashed lines indicate the pathways of the visual targets

**Fig. 3:**
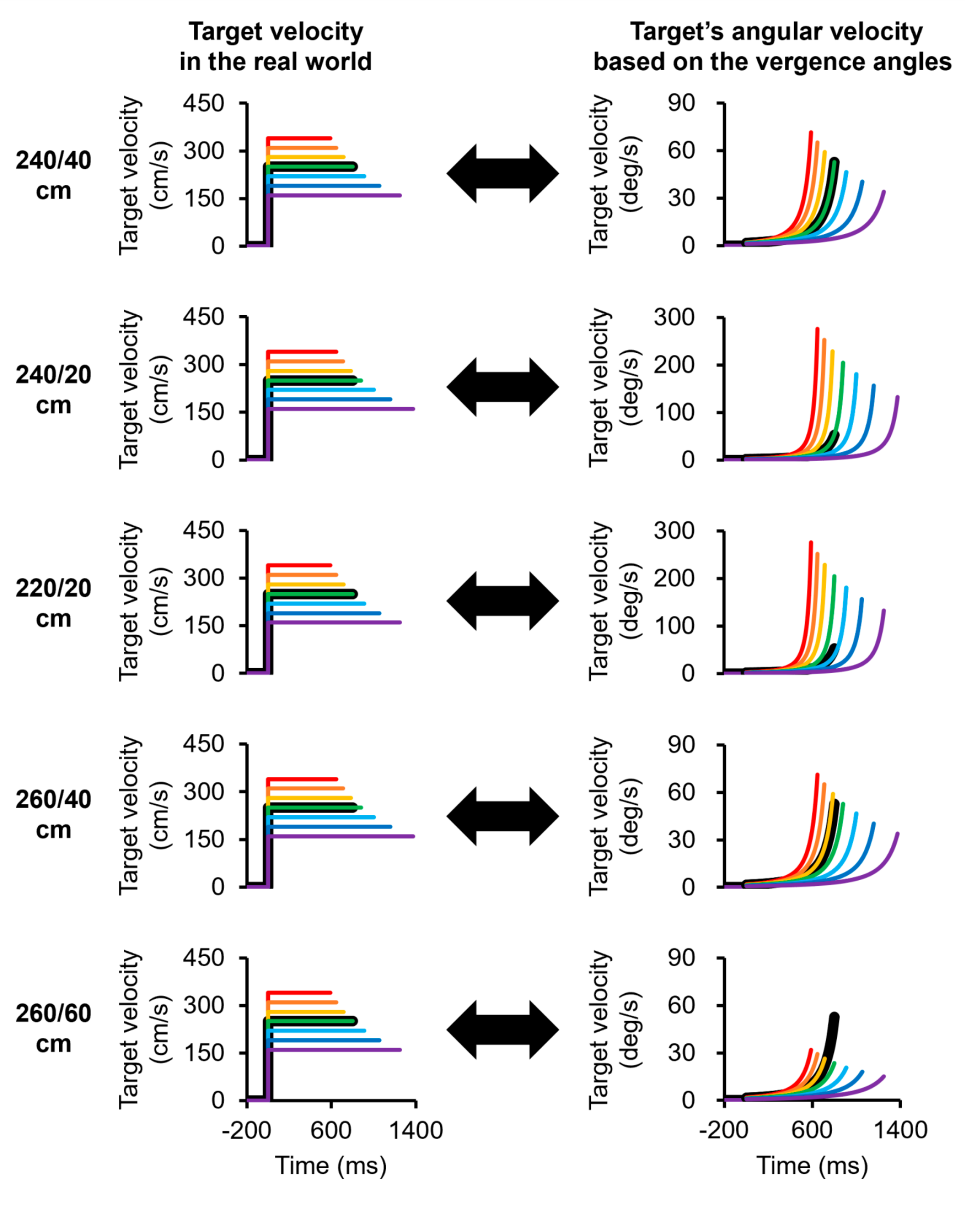
Target velocity in the real world and the angular velocity of the target derived from the vergence angles in Experiment 1. Stimulus I (240/40 cm), Stimulus II (240/20 cm), Stimulus III (220/20 cm), Stimulus IV (260/40 cm) and Stimulus V (260/60 cm) are shown. The target moves at a constant speed in the real world (physically), while it moves with acceleration in terms of the angular velocity. In the Stimulus I condition, 240/40 cm means that the target approaches from a distance of 240 cm away from the observer to a distance of 40 cm from the observer, and the same applies to the other stimuli. The red, orange, yellow, green, aqua, blue and purple lines indicate the order of speed. The black lines indicate the standard stimuli

For this experimental task, we used 5 types of stimuli, consisting of moving visual targets with different initial or final positions (Fig. 2). The first visual target moved from a distance of 240 cm to 40 cm away from a participant (Stimulus I), equivalent to vergence angles of 1.4 to 8.6 deg. In this study, the vergence angles were calculated by assigning the interocular distance as 6.25 cm (Nefs & Harris, 2007). The second visual target moved from 240 cm to 20 cm away from the participant (Stimulus II), equivalent to vergence angles of 1.4 to 17.1 deg. The third visual target moved from 220 cm to 20 cm away from the participant (Stimulus III), equivalent to vergence angles of 1.6 to 17.1 deg. The fourth visual target moved from 260 cm to 40 cm away from the participant (Stimulus IV), equivalent to vergence angles of 1.3 to 8.6 deg. The final visual target moved from 260 cm to 60 cm away from the participant (Stimulus V), equivalent to vergence angles of 1.3 to 5.7 deg. In the Stimulus I and II conditions, the initial position of the visual target was set at a distance of 240 cm from the observer to verify the velocity perception when the target started moving with a smaller rate of change in the angular velocity. In the Stimulus I and II conditions, the final position was set at a distance of 40 cm–20 cm from the observer. These positions were selected because a target approaching at a distance of 20 cm from the observer was perceived as faster than a target approaching at a distance of 40 cm from the observer in our previous study (Yoshimura et al., 2023). In the Stimulus III, IV and V conditions, the initial position of the visual target was set at a distance of 220–260 cm from the observer, and the final position of the visual target was set at a distance of 20, 40–60 cm from the observer. These positions were selected because Stimuli I and II were different not only in terms of their final position but also in terms of the moving distance and duration. The information on the moving distance and duration could be used to perceive the target velocity and determine temporal information (Cohen et al., 1953; Jones & Huang, 1982). Thus, we used Stimuli III, IV and V with corresponding moving distances (200–220 cm) in addition to Stimuli I and II. For each stimulus, the visual target moved at one of seven velocities (160, 190, 220, 250, 280, 310–340 cm/s). Figure 3 shows all the target velocities in the real world and the angular velocity of the target derived from the vergence angles for each stimulus type.

In Experiment 1, we compared the perceived velocity in the Stimulus II, III, IV, and V conditions with that in the Stimulus I condition on the basis of the following three hypotheses. The first hypothesis is that if velocity perception relies on a greater change in angular velocity or the threat of a target approaching the observer, Stimulus II and Stimulus III would be perceived as faster than Stimulus I, which is similar to the results of our previous study (Yoshimura et al., 2023). The second hypothesis is that if the perceived velocity depends on the initial phase of target motion, there would be little difference between the perceived velocities of Stimuli II, III, IV, and V and that of the Stimulus I. This is because the rate of change in the angular velocity for the initial phase of those stimuli is so small that the angular velocity of the stimuli is only slightly different. The third hypothesis is that if a stimulus with a longer duration or moving distance is perceived as faster, Stimulus II and Stimulus IV would be perceived as faster than Stimulus I. Previous studies have reported that a faster moving target is perceived as having a longer temporal duration than a slower moving target (Brown, 1995; Kanai et al., 2006; Kaneko and Murakami, 2009a; Makin et al., 2012). Thus, if the converse of the reports of the previous studies is true, Stimulus II and Stimulus IV, which have longer durations than Stimulus I, would be perceived as faster than Stimulus I.

#### Experimental procedures

**Fig. 4:**
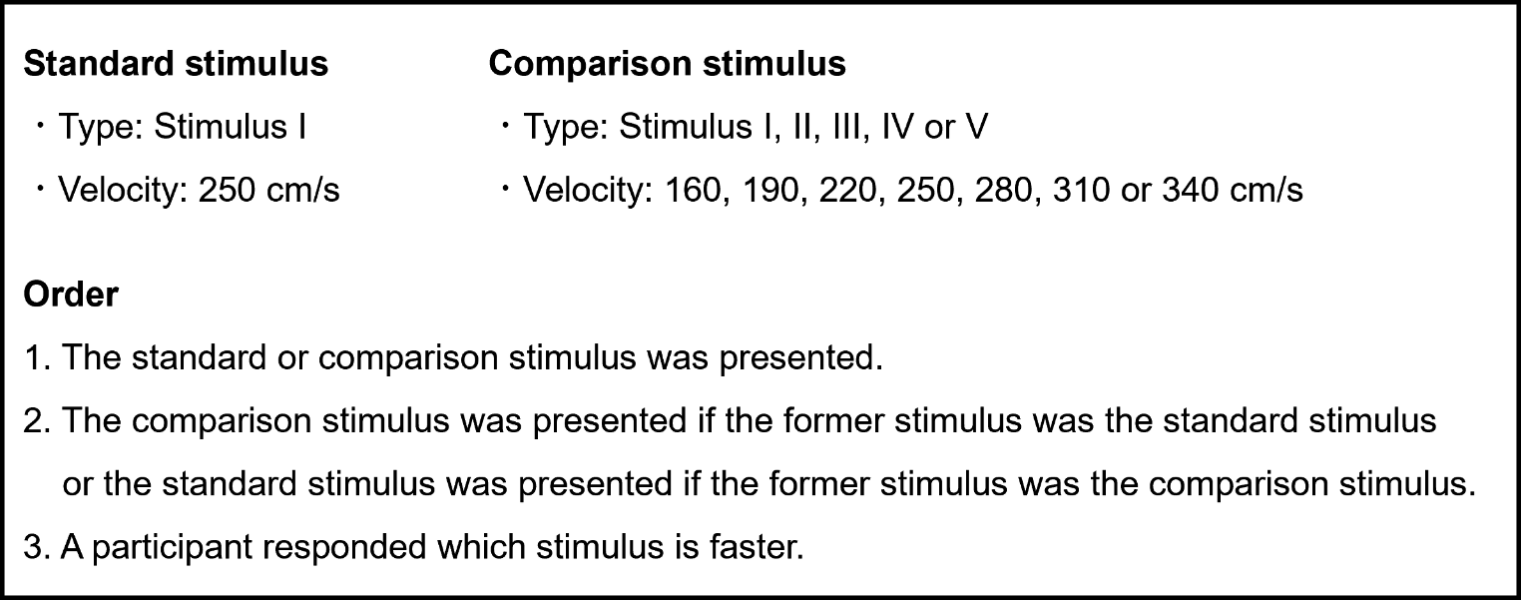
Outline of the experimental task in Experiment 1

The participants were seated with their head stabilized by a chin rest and a forehead restraint in a dimly lit room so that the vertical distance from their eyes to the horizontal screen was 5 cm. We measured speed perception via a two-alternative forced-choice (2AFC) paradigm. The participants were asked to respond by pressing a keyboard to indicate whether the speed of the latter stimulus was perceived as faster or slower than that of the former stimulus after they tracked a sequence of stimuli with their eyes (Fig. 4). For a sequence of stimuli, one stimulus was a standard stimulus, which was always a visual target moving at 250 cm/s in the Stimulus I condition, and the other stimulus was a comparison stimulus, which was one of 5 types of stimuli with 7 velocities. The participants performed 20 blocks of 35 trials (5 types of stimuli × 7 velocities), with a total of 700 trials. The participants also performed this experimental task on two or three separate days, considering the effects of fatigue and burden.

#### Data and statistical analyses

In the 2AFC paradigm, the probability that a participant indicated that the comparison stimulus was faster than the standard stimulus was calculated for each target velocity for each type of stimulus. Then, psychometric functions were derived from the probabilities of each type of stimulus via binomial logistic regression analysis, and the coefficient of determination (R^2^) for each psychometric function was obtained to analyze goodness of fit. Moreover, if the coefficients of the model estimated from the regression analysis were statistically significant (*p* < 0.05), the point of subjective equality (PSE) was calculated on the basis of the psychometric function. In this study, we defined the PSE as the speed at the 50% point on the fitted psychometric function. We also performed the bootstrap method with the psychometric function 1000 times to calculate the two-sided 95% confidence interval of the PSE. These analyses were conducted via MATLAB R2024a (MathWorks Inc., MA).

To compare the PSEs of Stimuli II, III, IV, and V against that of Stimulus I, which was always used as the standard stimulus, we conducted a Bayesian paired t test. We also calculated Bayes factors (BF_10_), which indicate the ratio of the likelihood of the alternative hypothesis compared with the null hypothesis, to verify the plausibility of the null or alternative hypothesis (van Doorn et al., 2021). The prior for the Bayesian paired t test was set on the basis of the Cauchy distribution with a width of 0.7, which was the default prior for that test in JASP statistical software ver. 0.18.3.0 (AMS). The evaluation of BF_10_ was performed on the basis of Jeffreys (1961) and Lee and Wagenmakers (2013) (Jeffreys, 1961; Lee & Wagenmakers, 2013).

### Results

**Fig. 5:**
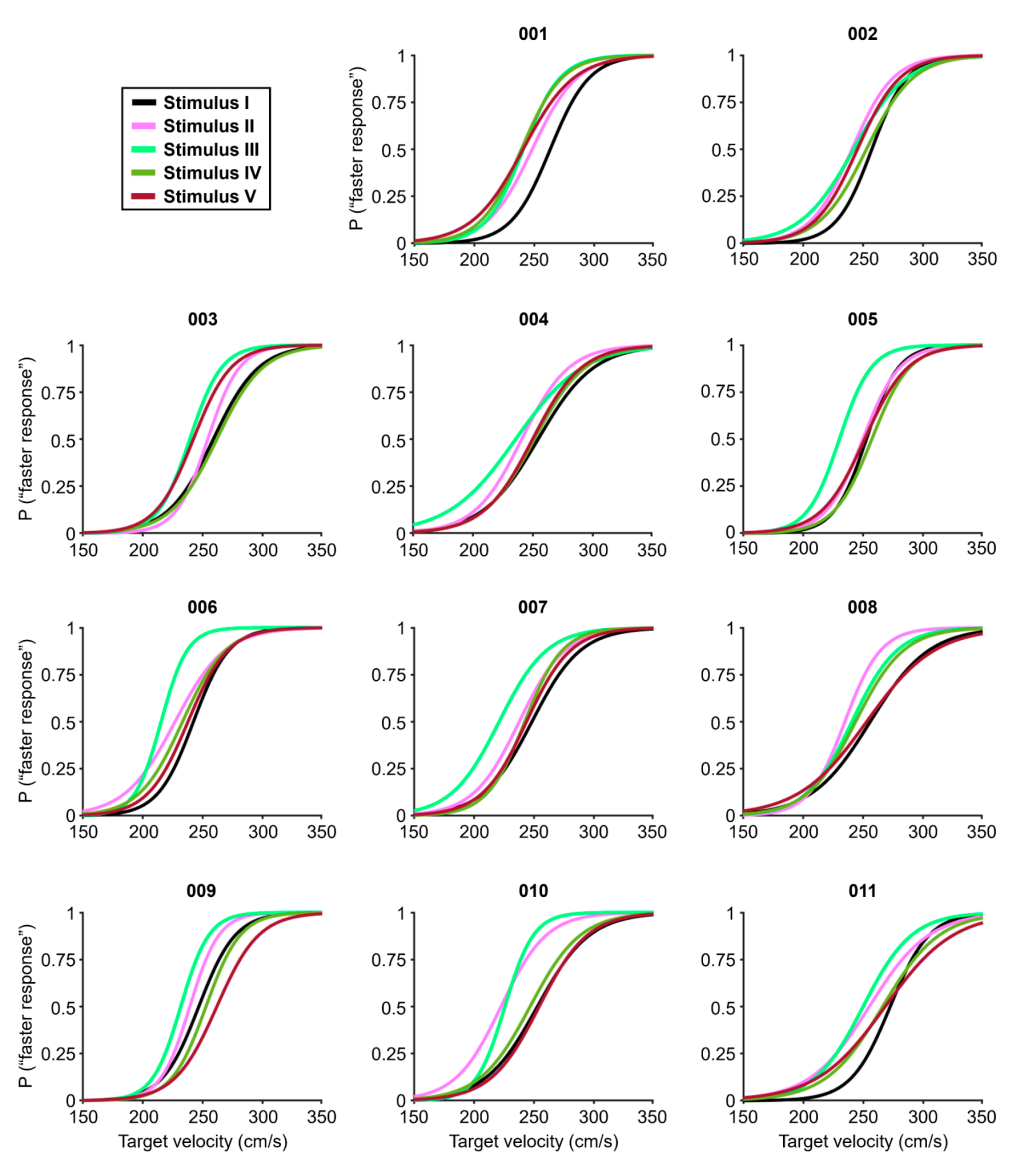
The psychometric functions for 5 types of stimuli for each participant in Experiment 1, where 001–011 represent the participants; P (“faster response”) indicates the probability that the comparison stimulus was perceived as faster than the standard stimulus

**Fig. 6:**
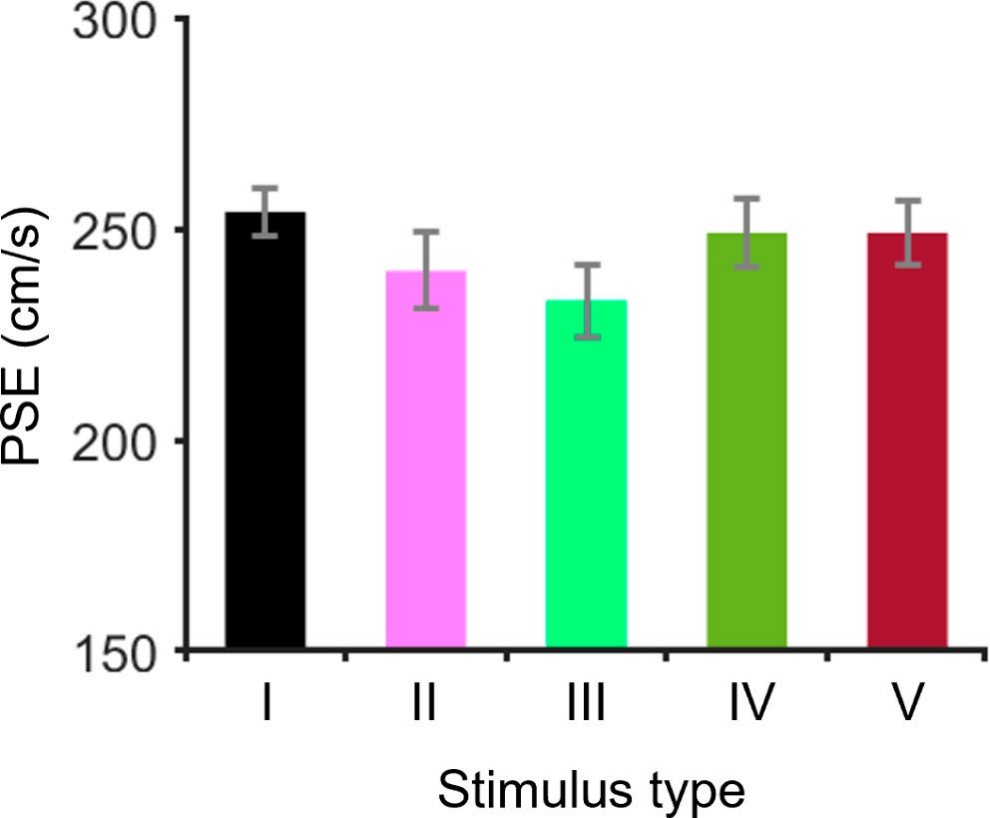
The PSEs for each stimulus in Experiment 1. The error bars indicate the standard deviation

Figure 5 and Electronic Supplementary Material (ESM) 1 show the psychometric functions of each participant for the five types of stimuli. The mean values (± SDs) of the PSE for all participants for Stimuli I, II, III, IV, and V were 254.0 ± 6.3, 240.2 ± 9.3,233.1 ± 8.9, 249.2 ± 9.0 and 249.4 ± 8.1 cm/s, respectively (Fig. 6). On the basis of the PSE results in the Bayesian paired t test, the BF_10_ for Stimulus II against Stimulus I was 223, which extremely supports the alternative hypothesis. The BF_10_ for Stimulus III against Stimulus I was 334,278, which extremely supports the alternative hypothesis. The BF_10_ for Stimulus IV against Stimulus I was 1.01, which indicates anecdotal support for the alternative hypothesis. The BF_10_ for Stimulus V against Stimulus I was 0.72, which indicates anecdotal support for the null hypothesis. These results imply that observers perceive an approaching target faster when the target moves closer to the observer, which supports our first hypothesis.

## Experiment 2

The results of Experiment 1 supported the first hypothesis that velocity perception relies on a larger change in angular velocity or the threat of a target approaching the observer. However, it remains uncertain whether the larger change in angular velocity or the threat more strongly affects velocity perception for depth motion. Therefore, in Experiment 2, we measured velocity perception using stimuli moving away from the observer to address this question.

### Methods

#### Participants

Ten participants (6 men and 4 women; 24.9 ± 2.1 years old) volunteered to take part in this experiment. They reported having normal or corrected-to-normal vision and no known neurological or oculomotor disorders. Written informed consent was obtained from all participants before their participation. This study was conducted in accordance with the 2013 Declaration of Helsinki, and all experimental protocols were approved by the Research Ethics Committee at the Faculty of Health and Sport Sciences, University of Tsukuba.

#### Stimulus design

**Fig. 7:**
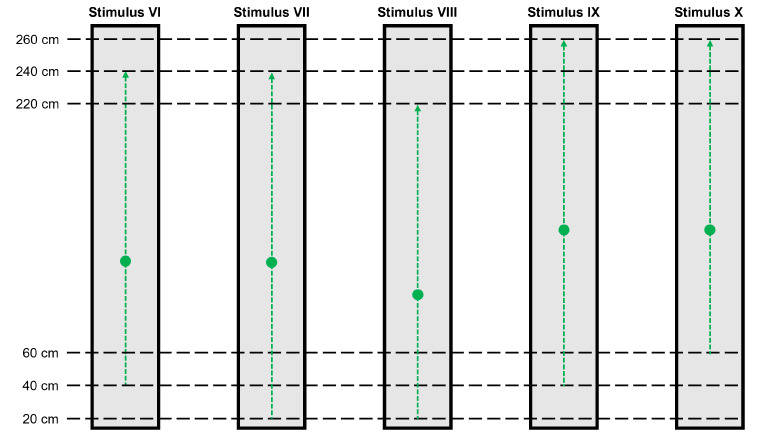
Moving distances of visual stimuli in Experiment 2. Stimuli VI, VII, VIII, IX, and X are shown. The black dashed lines indicate the distances from a participant in depth. The green circles indicate the visual targets. The green dashed lines indicate the pathways of the visual targets

**Fig. 8:**
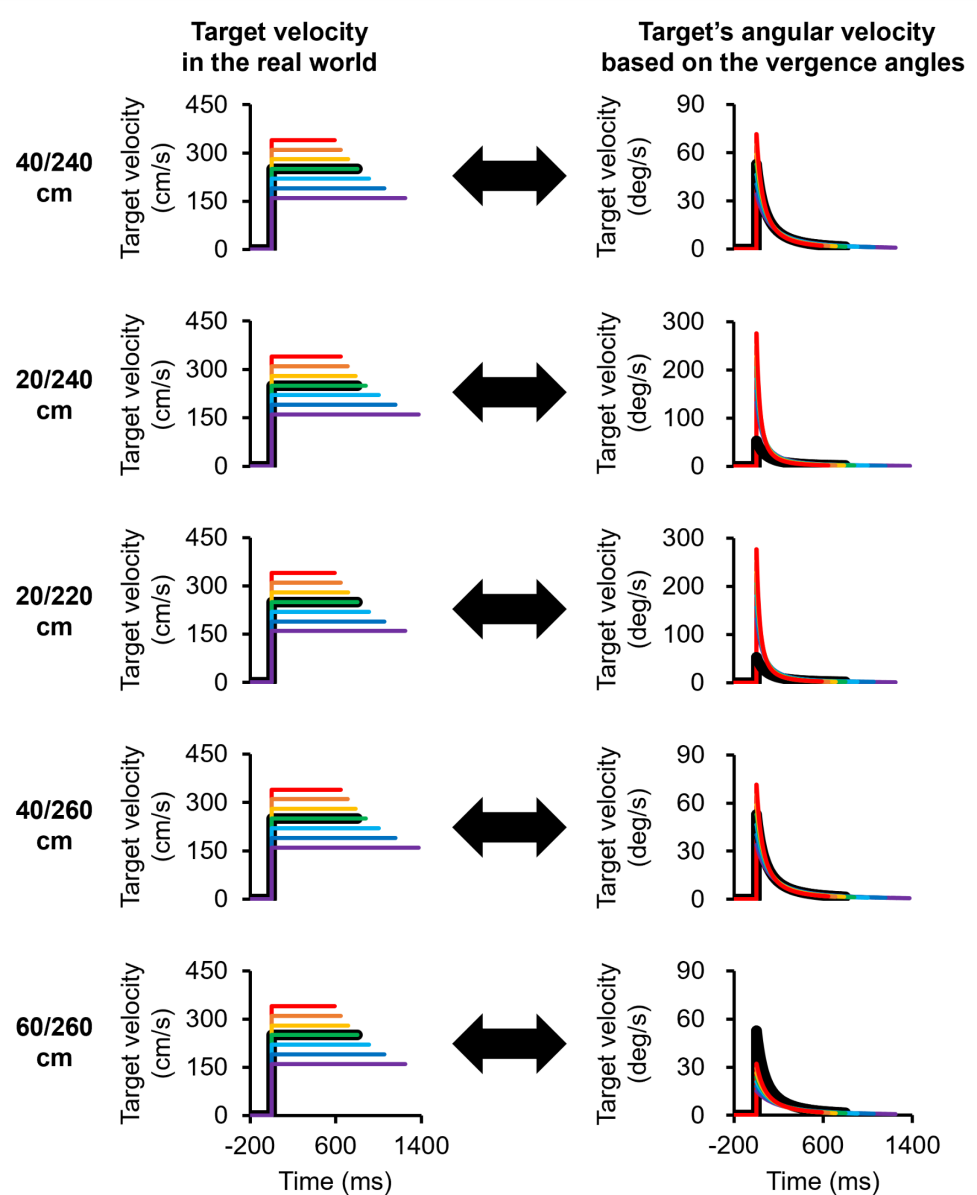
Target velocity in the real world and the angular velocity of the target derived from the vergence angles in Experiment 2. Stimulus VI (40/240 cm), Stimulus VII (20/240 cm), Stimulus VIII (20/220 cm), Stimulus IX (40/260 cm) and Stimulus X (60/260 cm) are shown. The target moves at a constant speed in the real world (physically), whereas it moves with deceleration in terms of the angular velocity. In the Stimulus VI condition, 40/240 cm means that the target moves from a distance of 40 cm from the observer to a distance of 240 cm, and the same applies to the other stimuli. The red, orange, yellow, green, aqua, blue, and purple lines indicate the order of speed. The black lines indicate the standard stimuli

The experimental setup in Experiment 2 was identical to that in Experiment 1 (Fig. 1). For the 5 types of stimuli in this experimental task, each stimulus moved in the opposite direction of each stimulus in Experiment 1 (Fig. 7). The first visual target moved from a distance of 40 cm to 240 cm from a participant (Stimulus VI). The second visual target moved from 20 cm to 240 cm from the participant (Stimulus VII). The third visual target moved from 20 cm to 220 cm from the participant (Stimulus VIII). The fourth visual target moved from 40 cm to 260 cm from the participant (Stimulus IX). The final visual target moved from 60 cm to 260 cm from the participant (Stimulus X). The target velocity conditions in this experiment were the same as those in Experiment 1 (160, 190, 220, 250, 280, 310–340 cm/s), but the angular velocities of the targets derived from the vergence angles were different in the two tasks (Fig. 8).

In Experiment 2, we compared the perceived velocity of Stimulus VII, VIII, IX or X against that of Stimulus VI on the basis of the two hypotheses. The first hypothesis is that if velocity perception relies on a greater change in angular velocity, Stimulus VII and Stimulus VIII would be perceived as faster than Stimulus VI. The other hypothesis is that if velocity perception relies on the threat of a target approaching the observer, there would be no difference between the perceived velocities of Stimuli VII, VIII, IX, and X and that of Stimulus VI. This is because our previous study revealed that the target velocity is perceived as faster only when the target approaches the observer and suggested that the angular velocity may not be a crucial factor for velocity perception, which means that the target velocity in the real world may affect velocity perception for depth motion (Yoshimura et al., 2023).

#### Experimental procedures, data and statistical analyses

**Fig. 9:**
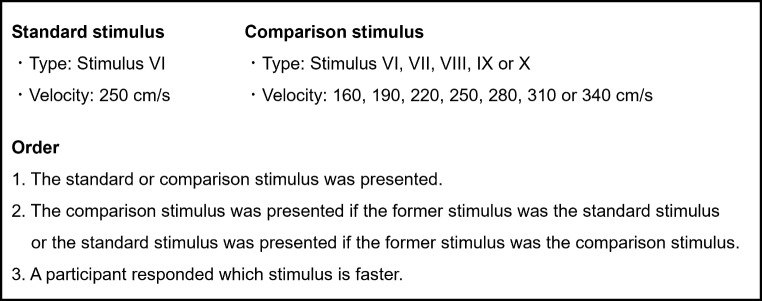
Outline of the experimental task in Experiment 2

The experimental procedures and data and statistical analyses used in this experiment were identical to those used in Experiment 1. In Experiment 2, however, the standard stimulus was Stimulus VI, and the comparison stimulus was Stimulus VI, VII, VIII, IX, or X (Fig. 9). The PSE of Stimulus VI (the standard stimulus) was compared with those of Stimuli VII, VIII, IX and X via the Bayesian paired t test.

### Results

**Fig. 10:**
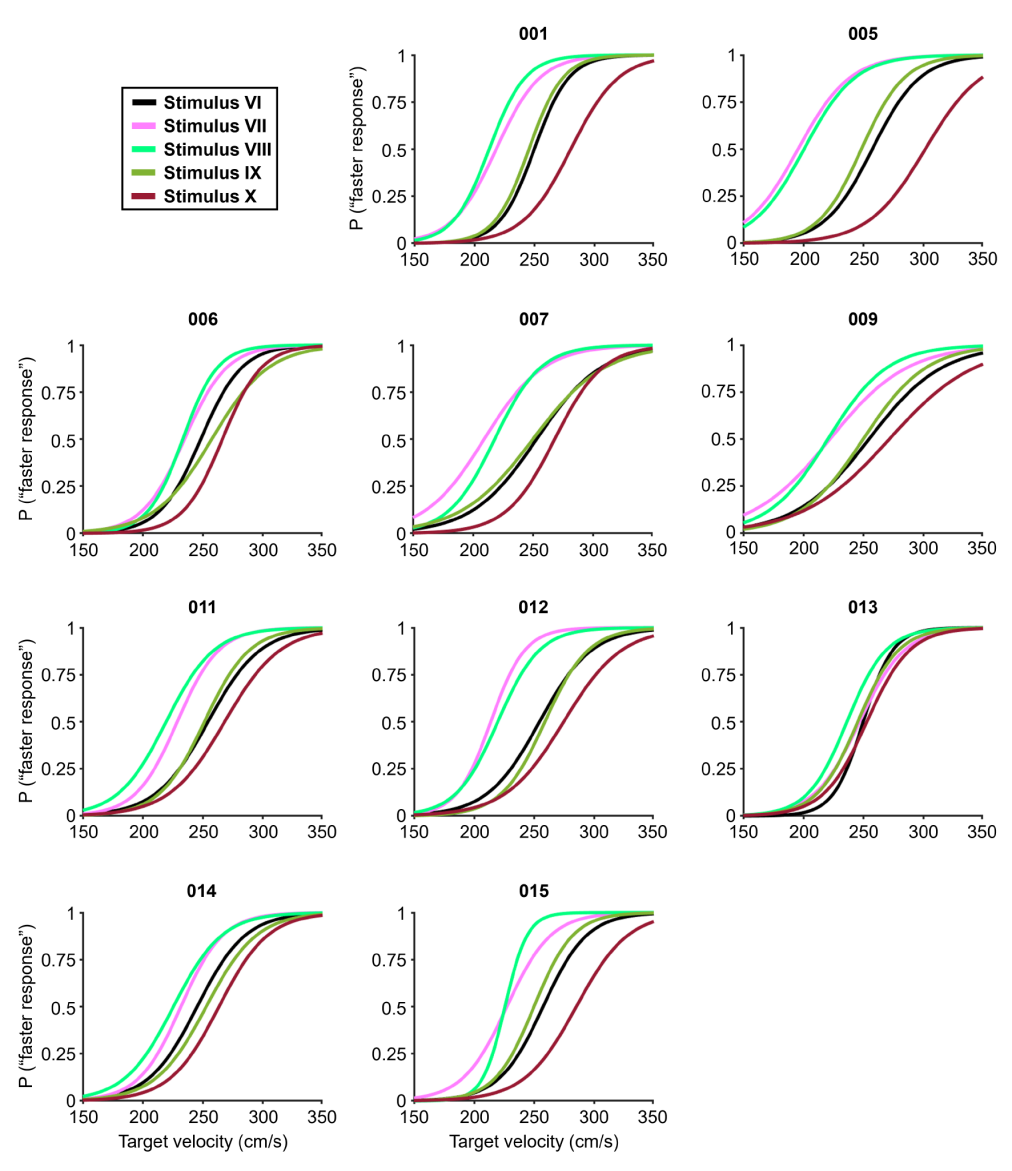
Psychometric functions for 5 types of stimuli for each participant in Experiment 2. The numbers (e.g., 001, 005) at the upper part of each chart represent the participants. P (“faster response”) indicates the probability that the comparison stimulus was perceived as faster than the standard stimulus

**Fig. 11:**
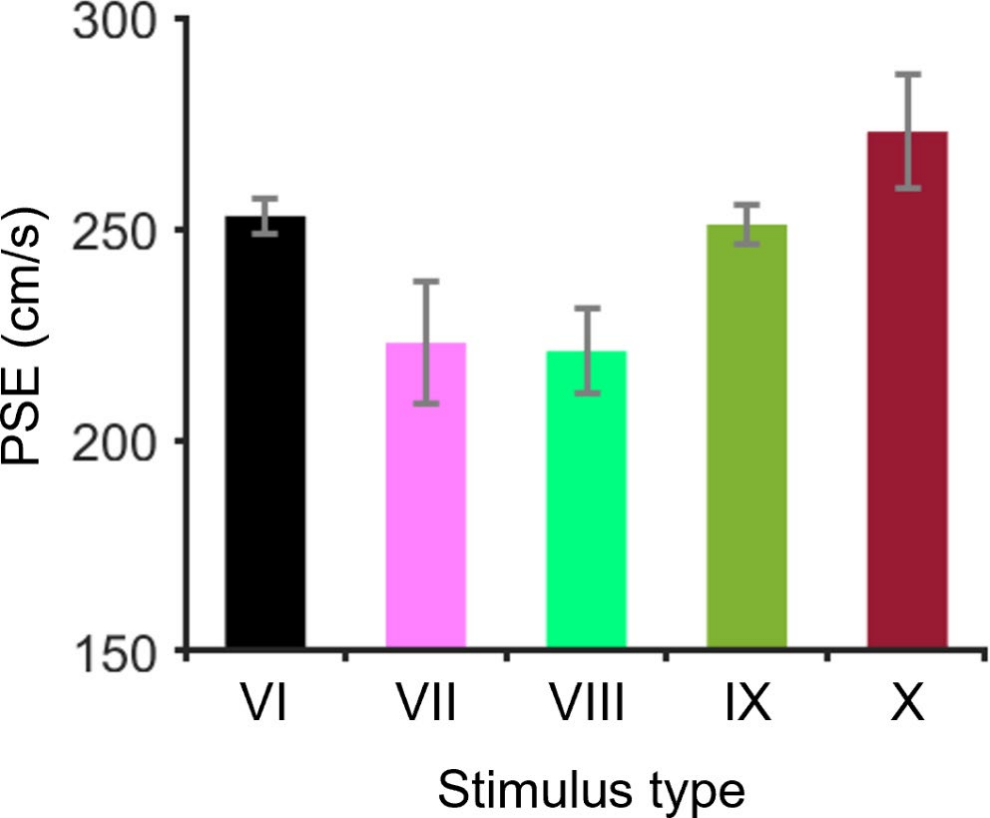
The PSEs for each stimulus in Experiment 2. The error bars indicate the standard deviation

Figure 10 and ESM 2 show the psychometric functions of each participant for the five types of stimuli. The mean values (± SDs) of the PSE for all participants for Stimuli VI, VII, VIII, IX, and X were 252.7 ± 4.3, 222.9 ± 14.5, 221.3 ± 10.1, 251.1 ± 4.6 and 273.4 ± 13.4 cm/s, respectively (Fig. 11). On the basis of the PSE results in the Bayesian paired t test, the BF_10_ for Stimulus VII against Stimulus VI was 88.3, which very strongly supports the alternative hypothesis. The BF_10_ for Stimulus VIII against Stimulus VI was 851, which extremely supports the alternative hypothesis. The BF_10_ for Stimulus IX against Stimulus VI was 0.4, which indicates anecdotal support for the null hypothesis. The BF_10_ for Stimulus X against Stimulus VI was 147, which extremely supports the alternative hypothesis. These results revealed that Stimulus VII and Stimulus VIII were perceived as faster than Stimulus VI was, and Stimulus X was perceived as slower than Stimulus VI was, which somewhat supports the first hypothesis for Experiment 2. Therefore, these results imply that observers perceive the target velocity for depth motion on the basis of larger changes in angular velocity.

## Discussion

In this study, we attempted to reveal the role of angular velocity based on the vergence angle and the initial phase of target motion in velocity perception for motion in the depth direction. In Experiment 1, the target was perceived as faster when the target approached the observer. In Experiment 2, even when the target moved away from the observer, the target was perceived as faster when the target started moving from a position closer to the observer. The results of both experiments suggest that targets moving in the depth direction are perceived as faster when the target is closer to the observer because the angular velocity increases steeply in this case. Therefore, the rate of change in the angular velocity plays a more important role in velocity perception for depth motion than does the initial phase of the target motion or other factors.

We considered that a larger change in the angular velocity of the target, which is based on the vergence angle, strongly affects velocity perception for motion in the depth direction. It has been reported that the firing rate of neurons in the extrastriate visual cortex transiently increases and then is sustained at a lower level after the onset of visual stimuli (Petersen et al., 1988). Neurons in the middle temporal (MT) area, which is associated with motion perception and is tuned to speed and direction (Britten et al., 1992; Maunsell & Van Essen, 1983; Newsome & Pare, 1988; Nichols & Newsome, 2002), also exhibit firing patterns similar to the neuronal activity described above when the velocity of the stimuli suddenly changes (Galashan et al., 2013; Lisberger & Movshon, 1999; Price & Born, 2010, 2013; Traschütz et al., 2015). Price and Born (2010) demonstrated that transients of MT neurons in response to speed changes were correlated with the perceptual decision of whether the stimulus speed was faster or slower (Price & Born, 2010). Moreover, transient activity of MT neurons in monkeys has been shown to correlate with human behavior in detecting sudden speed changes (Traschütz et al., 2015). Thus, neuronal activity in the MT area plays an important role in perceiving sudden speed changes. In the MT area, there are neurons tuned for binocular disparity (DeAngelis & Uka, 2003), which is a depth cue. Therefore, the observers in this study could detect larger changes in the angular velocity on the basis of the vergence angles, as shown in Figs. 3 and 8. Note that the velocity perception based on the larger change in the angular velocity of the target could be due not only to the vergence angles but also to the elevation angles or the larger change in the size of the image on the retina. This is because the elevation angles and image size, which are also depth cues, change similarly to the vergence angles (Brenner & Smeets, 2018; Gillam, 1995; McIntosh & Lashley, 2008; Ooi et al., 2001). The vergence angle, elevation angle and image size increase and decrease exponentially as the target approaches or moves away from the observer, respectively. It is necessary to examine the effects of these factors on the perceived velocity of motion in the depth direction in the future. However, in this study, the effect of changes in image size on velocity perception might have been less pronounced than the effect of larger changes in vergence angles. This is because the target on the screen was approximately a 10 mm circle, and the rate of change in the image size was relatively small.

Visual information on the initial phase of a moving target might also be informative for velocity perception, at least for depth motion. In Experiment 1, Stimulus V was not perceived as slower than Stimulus I was, whereas in Experiment 2, Stimulus X was perceived as slower than Stimulus VI was. These results cannot be explained by only the larger change in the angular velocity. Thus, it is possible that the difference in the results of the two experiments can be attributed to the visual information of the initial phase, as the difference in the angular velocity of the initial phase between Stimuli I and V in Experiment 1 was small, whereas that between Stimuli VI and X in Experiment 2 was large. It has been suggested that the initial phase of target motion influences the perceived velocity of the target in planar motion (Goettker et al., 2018, 2019). Because observers are more attentive at the onset of target motion (Abrams & Christ, 2003), visual information related to the initial phase of the target motion might be important in judging the target velocity. It has also been shown that the firing rate of MT neurons transiently increases at the onset of target motion (Lisberger & Movshon, 1999; Priebe et al., 2002; Priebe & Lisberger, 2002) and that MT neurons detect visual motion information within the first 75 ms after targets start moving (Pack & Born, 2001). Thus, information on the initial phase of the target motion is informative for velocity perception. In velocity perception for depth motion, it has been suggested that the initial phase of the target motion influences velocity perception; however, if the change in angular velocity based on the vergence angles exceeds the detection threshold for observers, the change in angular velocity more strongly affects velocity perception.

We found that the target motion was perceived as faster when the target position was closer to the observer, regardless of the moving direction. Here, we discuss whether the approaching stimuli in Experiment 1 are perceived as threats by the participants. Previous studies have investigated whether the time-to-contact estimation is different when threatening images, such as angry faces, snakes and spiders, and nonthreatening images, such as neutral faces, butterflies and rabbits, approach an observer (Brendel et al., 2012, 2014; DeLucia et al., 2014; Vagnoni et al., 2012). These studies have shown that the time-to-contact estimation is shorter for threatening images than for nonthreatening images. This result suggests that nonthreatening visual stimuli do not affect the perceived velocity of the target even if the target more closely approaches the observer. The approaching stimulus in Experiment 1 was a laser with a diameter of approximately 10 mm that was presented 5 cm below the observer’s eyes. Moreover, the stimulus stopped at least 20 cm from the observer. The stimuli in this experimental setup might not have been considered threats by the participants. Therefore, the targets approaching the observer more closely in our setup may not affect velocity perception.

However, the participants might choose their responses in the 2AFC paradigm on the basis of not only the target velocity but also other cues, which represents a limitation of the present study. Jogan and Stoker (2014) noted that perception in the standard 2AFC paradigm is affected by secondary stimulus attributes (e.g., stimulus noise, attention, or spatial context) (Jogan & Stocker, 2014). In this study, the participants had the ability to judge the target velocity according to where the target started and ended or by its duration or distance when it was uncertain which stimulus was moving faster in the 2AFC paradigm. However, this possibility can be ruled out in this experiment for the following reasons. First, the participants were not informed how many types of stimuli were included in the 2AFC paradigm before the experimental task to reduce the influence of secondary stimulus attributes as much as possible. Second, for stimulus duration or distance, our results showed that the velocities of Stimuli IV and IX were perceived to be the same as those of Stimuli I and VI, respectively, suggesting that duration does not influence velocity perception. This finding is not consistent with previous studies showing that the duration of a moving target affects temporal judgments (Brown, 1995; Cohen et al., 1953; Jones & Huang, 1982; Kanai et al., 2006; Kaneko & Murakami, 2009b; Li et al., 2015; Makin et al., 2012). This suggests that the effect of a greater change in the angular velocity of the target on velocity perception was greater than the effect of duration or distance. Therefore, in this study, velocity perception was most likely influenced more by larger changes in the angular velocity of the target than by secondary stimulus attributes.

## Conclusion

We aimed to reveal the role of the angular velocity derived from the vergence angle and the initial phase of target motion in velocity perception for depth motion. Our results showed that when the target moved toward the observer, the target was perceived as moving faster when the target was closer to the observer. Our results also showed that when the target moved away from the observer, the closer the initial position of the target was, the faster the perceived velocity of the target. Our findings suggest that greater changes in angular velocity could be important in velocity perception for motion in the depth direction, regardless of visual information on the initial phase of the target motion. Therefore, observers perceive the target velocity as faster when the target is closer to the observer.

## Electronic supplementary material

Below is the link to the electronic supplementary material.

Electronic Supplementary Material

## Data Availability

The data and materials are available from the corresponding author upon request.
