# Supplementary material for: Motion in the depth direction appears faster when the target is closer to the observer
Source: Psychol Res. 2024 Nov 20;89(1):25. doi: 10.1007/s00426-024-02040-w (PMC11579063; doi:10.1007/s00426-024-02040-w)
Supplement: Supplementary file 1 — Electronic Supplementary Material [file 426_2024_2040_MOESM1_ESM.pdf]

Article title: Motion in the depth direction appears faster when the target is closer to the observer

Journal name: Psychological Research

Author names and affiliations:

Yusei Yoshimura

Faculty of Health and Sport Sciences, University of Tsukuba

1-1-1, Tennodai, Tsukuba, Ibaraki 305-8574, Japan

Tomohiro Kizuka

Faculty of Health and Sport Sciences, University of Tsukuba

1-1-1, Tennodai, Tsukuba, Ibaraki 305-8574, Japan

Seiji Ono (corresponding author)

Faculty of Health and Sport Sciences, University of Tsukuba

1-1-1, Tennodai, Tsukuba, Ibaraki 305-8574, Japan

Email: ono.seiji.fp@u.tsukuba.ac.jp

Electronic supplementary material (ESM)

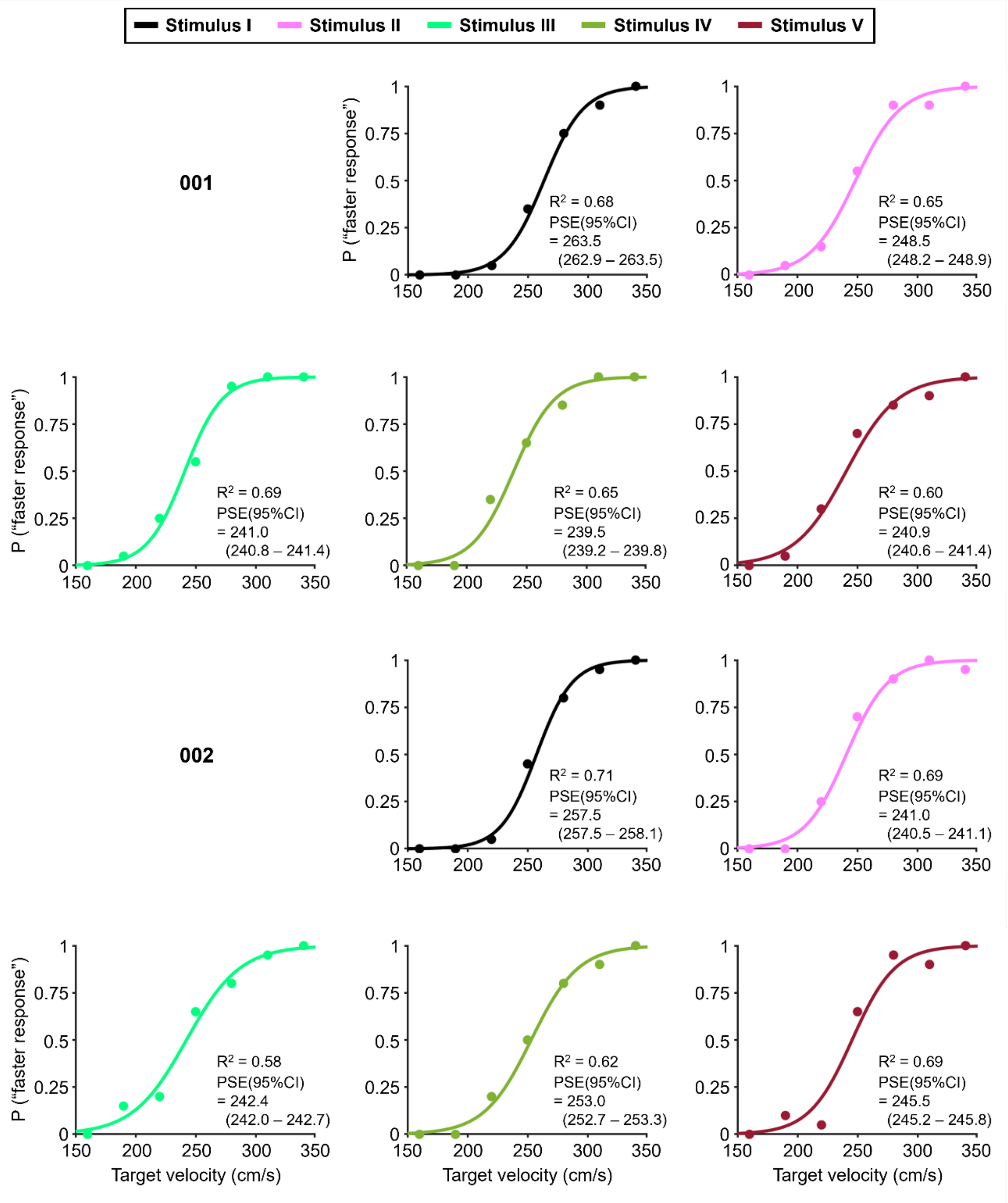

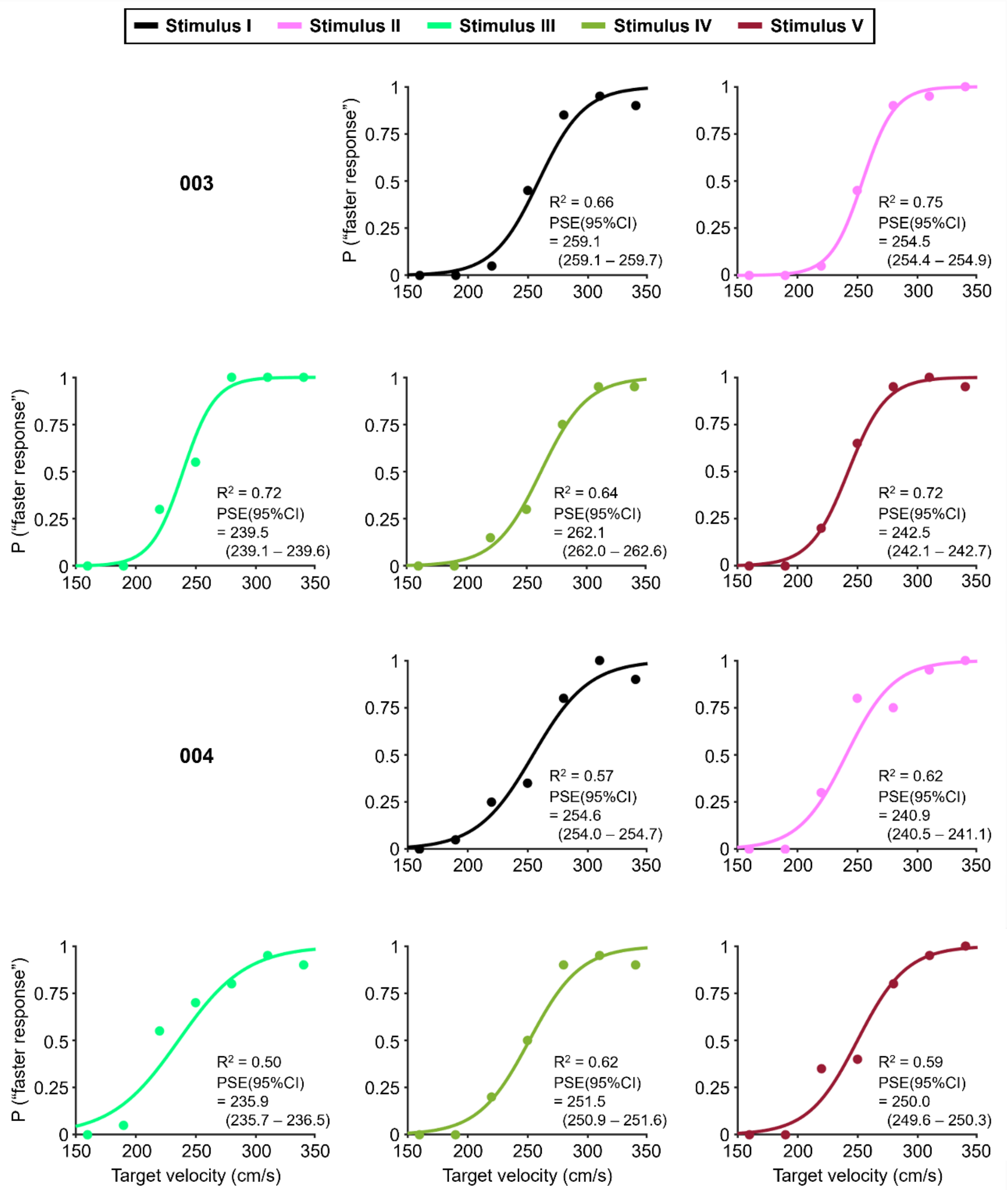
Stimulus I
Stimulus II
Stimulus III
Stimulus IV
Stimulus V
003
004
P ("faster response")
Target velocity (cm/s)
R² = 0.66 PSE(95%CI) = 259.1 (259.1 – 259.7)
R² = 0.75 PSE(95%CI) = 254.5 (254.4 – 254.9)
R² = 0.72 PSE(95%CI) = 239.5 (239.1 – 239.6)
R² = 0.64 PSE(95%CI) = 262.1 (262.0 – 262.6)
R² = 0.72 PSE(95%CI) = 242.5 (242.1 – 242.7)
R² = 0.57 PSE(95%CI) = 254.6 (254.0 – 254.7)
R² = 0.62 PSE(95%CI) = 240.9 (240.5 – 241.1)
R² = 0.50 PSE(95%CI) = 235.9 (235.7 – 236.5)
R² = 0.62 PSE(95%CI) = 251.5 (250.9 – 251.6)
R² = 0.59 PSE(95%CI) = 250.0 (249.6 – 250.3)

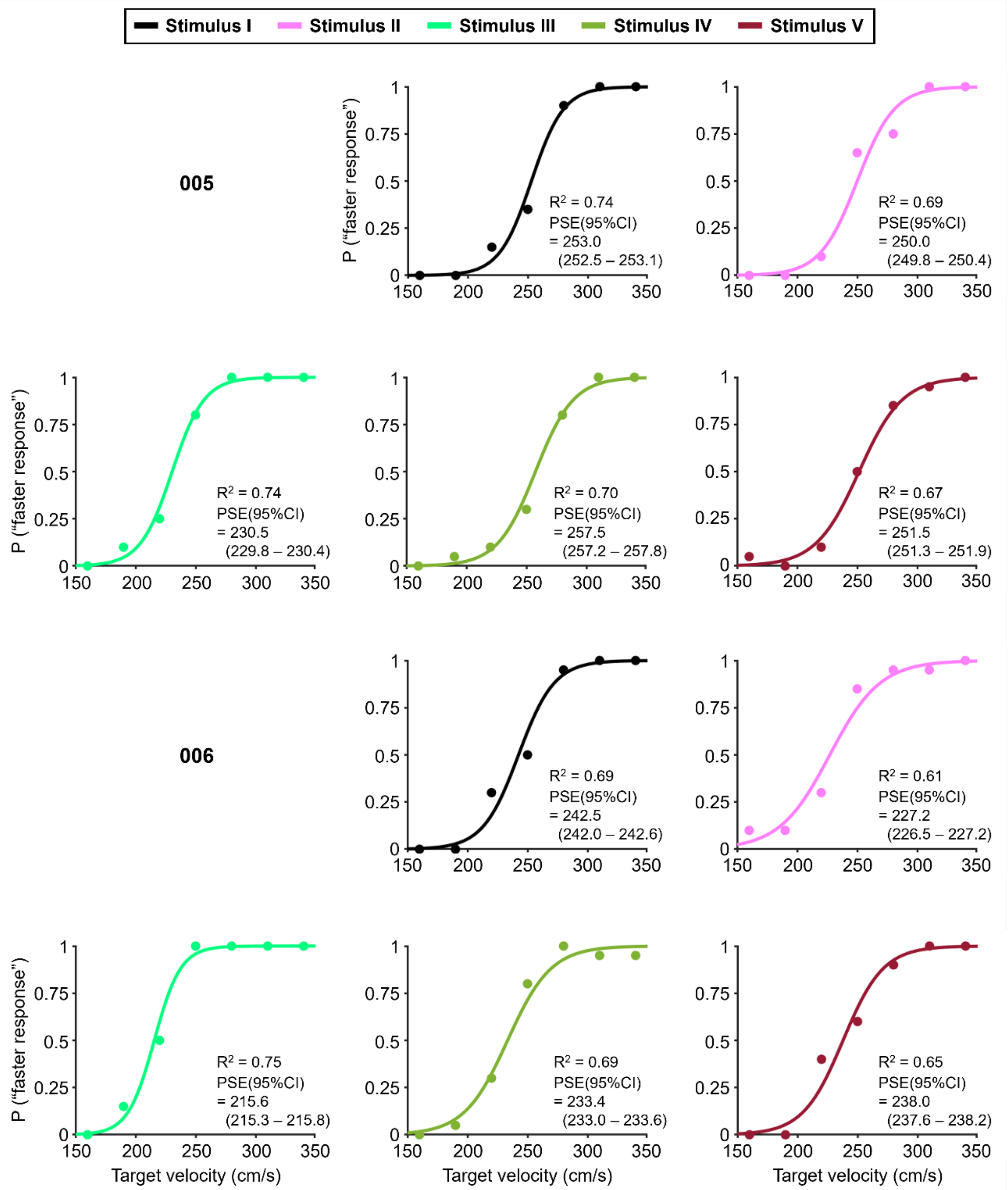

Stimulus I
Stimulus II
Stimulus III
Stimulus IV
Stimulus V
005
006
P (“faster response”)
Target velocity (cm/s)
R² = 0.74 PSE(95%CI) = 253.0 (252.5 – 253.1)
R² = 0.69 PSE(95%CI) = 250.0 (249.8 – 250.4)
R² = 0.74 PSE(95%CI) = 230.5 (229.8 – 230.4)
R² = 0.70 PSE(95%CI) = 257.5 (257.2 – 257.8)
R² = 0.67 PSE(95%CI) = 251.5 (251.3 – 251.9)
R² = 0.69 PSE(95%CI) = 242.5 (242.0 – 242.6)
R² = 0.61 PSE(95%CI) = 227.2 (226.5 – 227.2)
R² = 0.75 PSE(95%CI) = 215.6 (215.3 – 215.8)
R² = 0.69 PSE(95%CI) = 233.4 (233.0 – 233.6)
R² = 0.65 PSE(95%CI) = 238.0 (237.6 – 238.2)

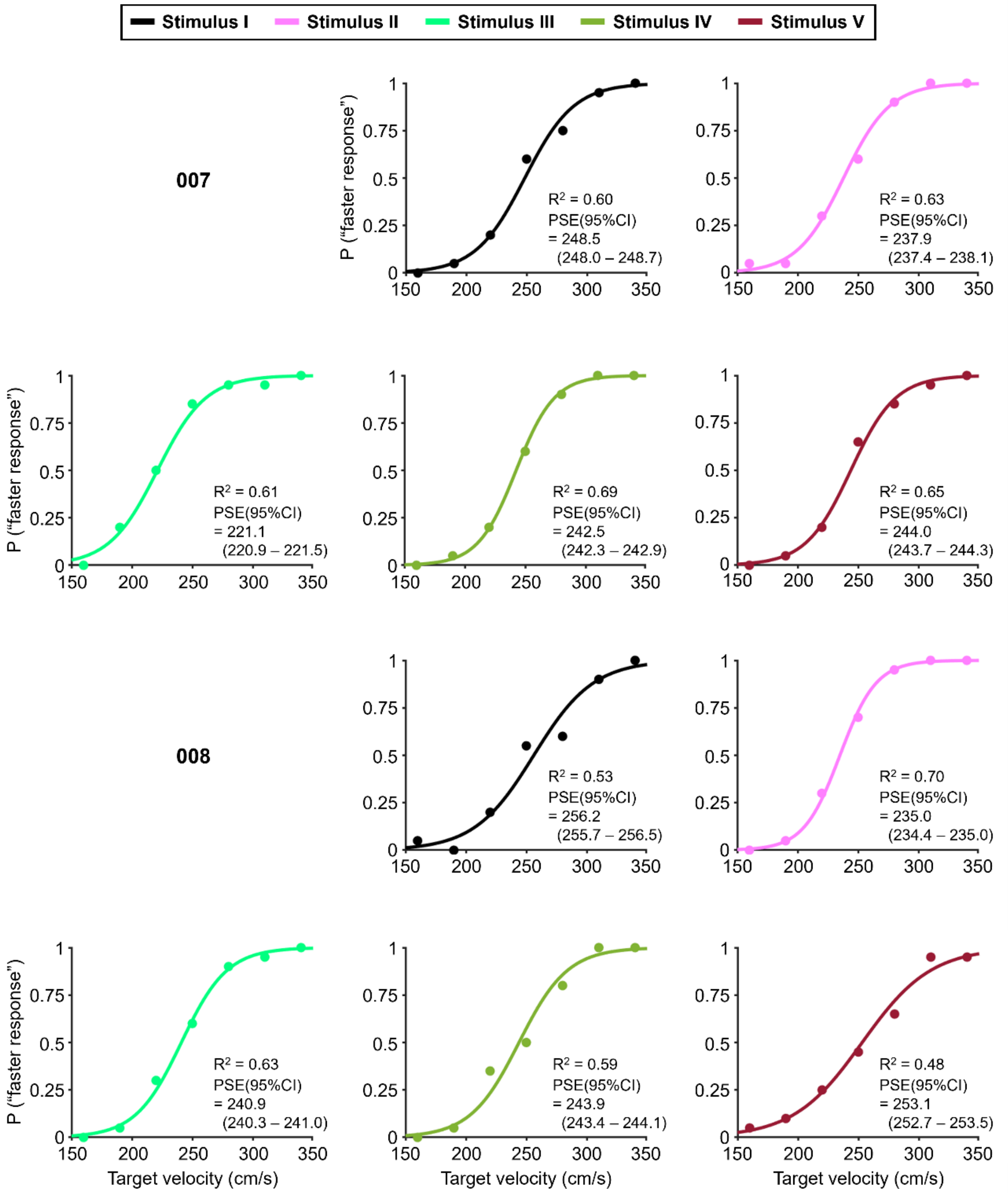

Stimulus I
Stimulus II
Stimulus III
Stimulus IV
Stimulus V
007
008
P ("faster response")
Target velocity (cm/s)
R² = 0.60 PSE(95%CI) = 248.5 (248.0 – 248.7)
R² = 0.63 PSE(95%CI) = 237.9 (237.4 – 238.1)
R² = 0.61 PSE(95%CI) = 221.1 (220.9 – 221.5)
R² = 0.69 PSE(95%CI) = 242.5 (242.3 – 242.9)
R² = 0.65 PSE(95%CI) = 244.0 (243.7 – 244.3)
R² = 0.53 PSE(95%CI) = 256.2 (255.7 – 256.5)
R² = 0.70 PSE(95%CI) = 235.0 (234.4 – 235.0)
R² = 0.63 PSE(95%CI) = 240.9 (240.3 – 241.0)
R² = 0.59 PSE(95%CI) = 243.9 (243.4 – 244.1)
R² = 0.48 PSE(95%CI) = 253.1 (252.7 – 253.5)

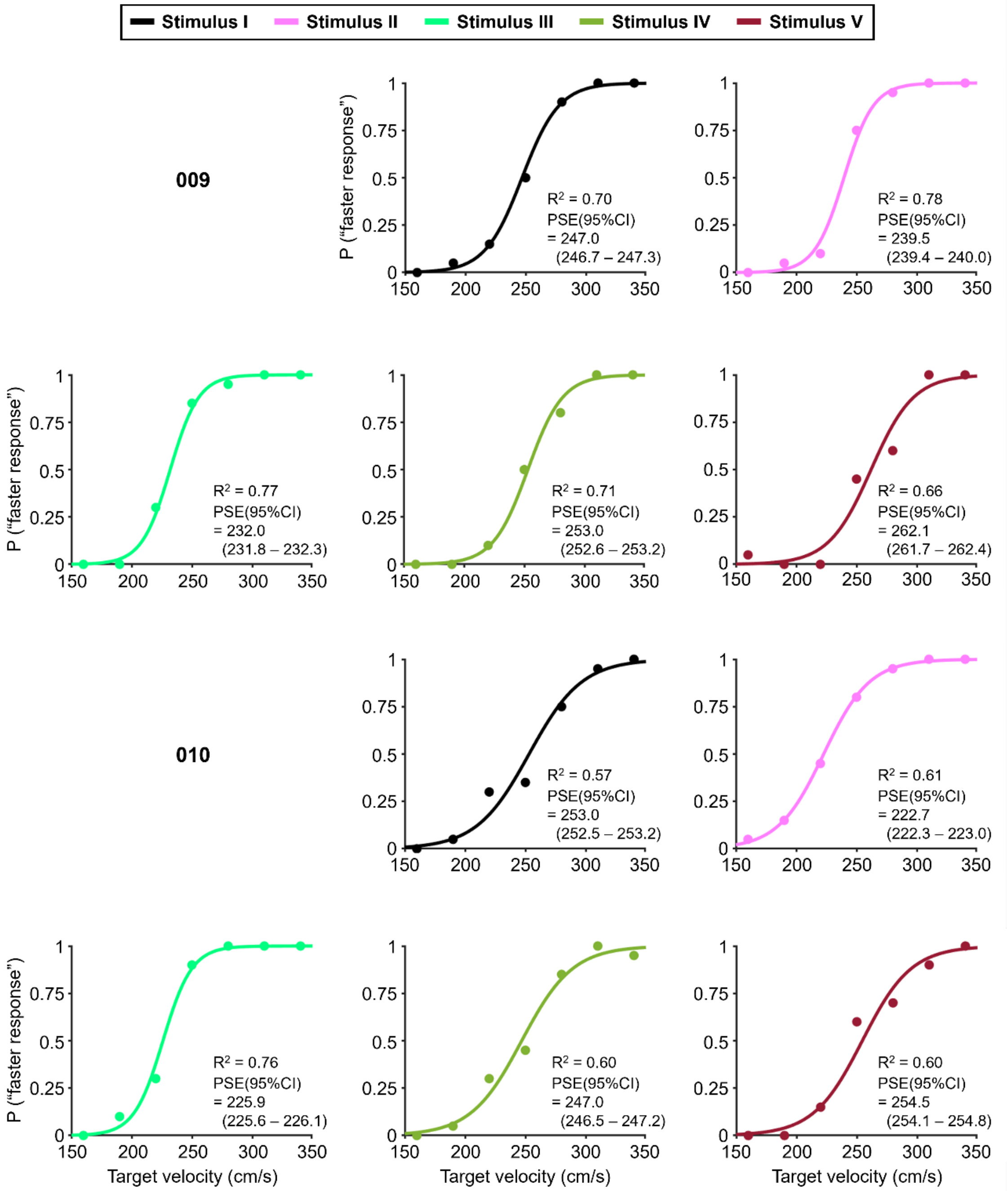

Stimulus I
Stimulus II
Stimulus III
Stimulus IV
Stimulus V
009
010
P (“faster response”)
Target velocity (cm/s)
R² = 0.70 PSE(95%CI) = 247.0 (246.7 – 247.3)
R² = 0.78 PSE(95%CI) = 239.5 (239.4 – 240.0)
R² = 0.77 PSE(95%CI) = 232.0 (231.8 – 232.3)
R² = 0.71 PSE(95%CI) = 253.0 (252.6 – 253.2)
R² = 0.66 PSE(95%CI) = 262.1 (261.7 – 262.4)
R² = 0.57 PSE(95%CI) = 253.0 (252.5 – 253.2)
R² = 0.61 PSE(95%CI) = 222.7 (222.3 – 223.0)
R² = 0.76 PSE(95%CI) = 225.9 (225.6 – 226.1)
R² = 0.60 PSE(95%CI) = 247.0 (246.5 – 247.2)
R² = 0.60 PSE(95%CI) = 254.5 (254.1 – 254.8)

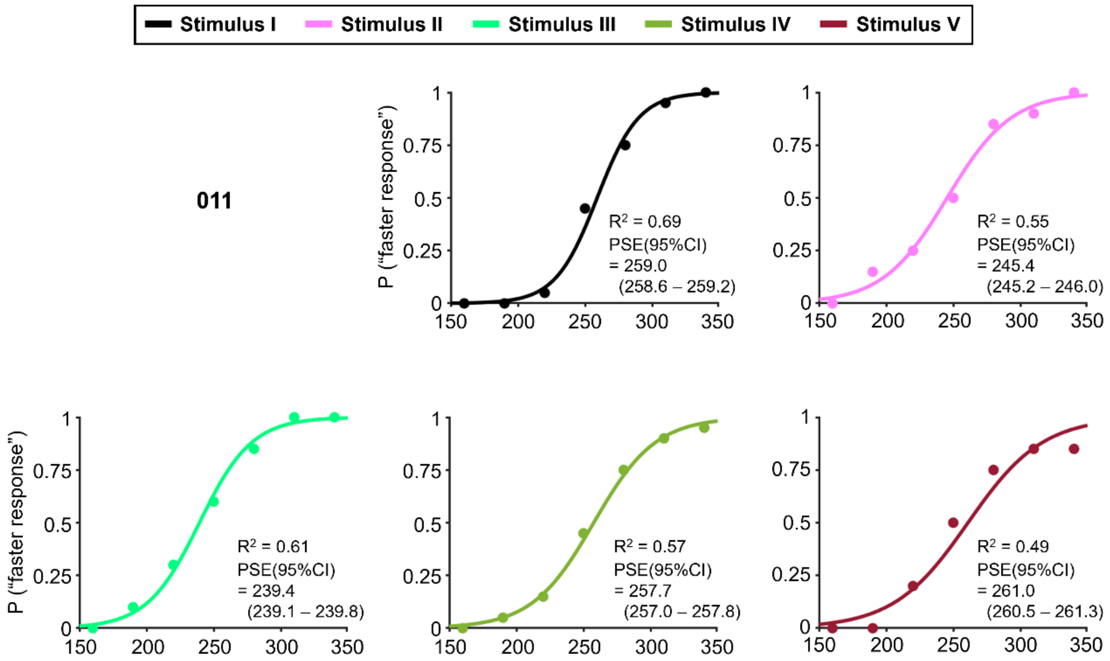

ESM 1 The psychometric functions in each stimulus for each participant in Experiment 1. 001 - 011 represent the participants. Each dot shows the probability that a participant indicated that the comparison stimulus for each target velocity was faster than the standard stimulus. The R^2 indicates the coefficient of determination of psychometric functions. The PSE(95%CI) represents the point of subjective equality based on the psychometric function (the lower bound - the upper bound in 95 % confidence interval of the PSE).

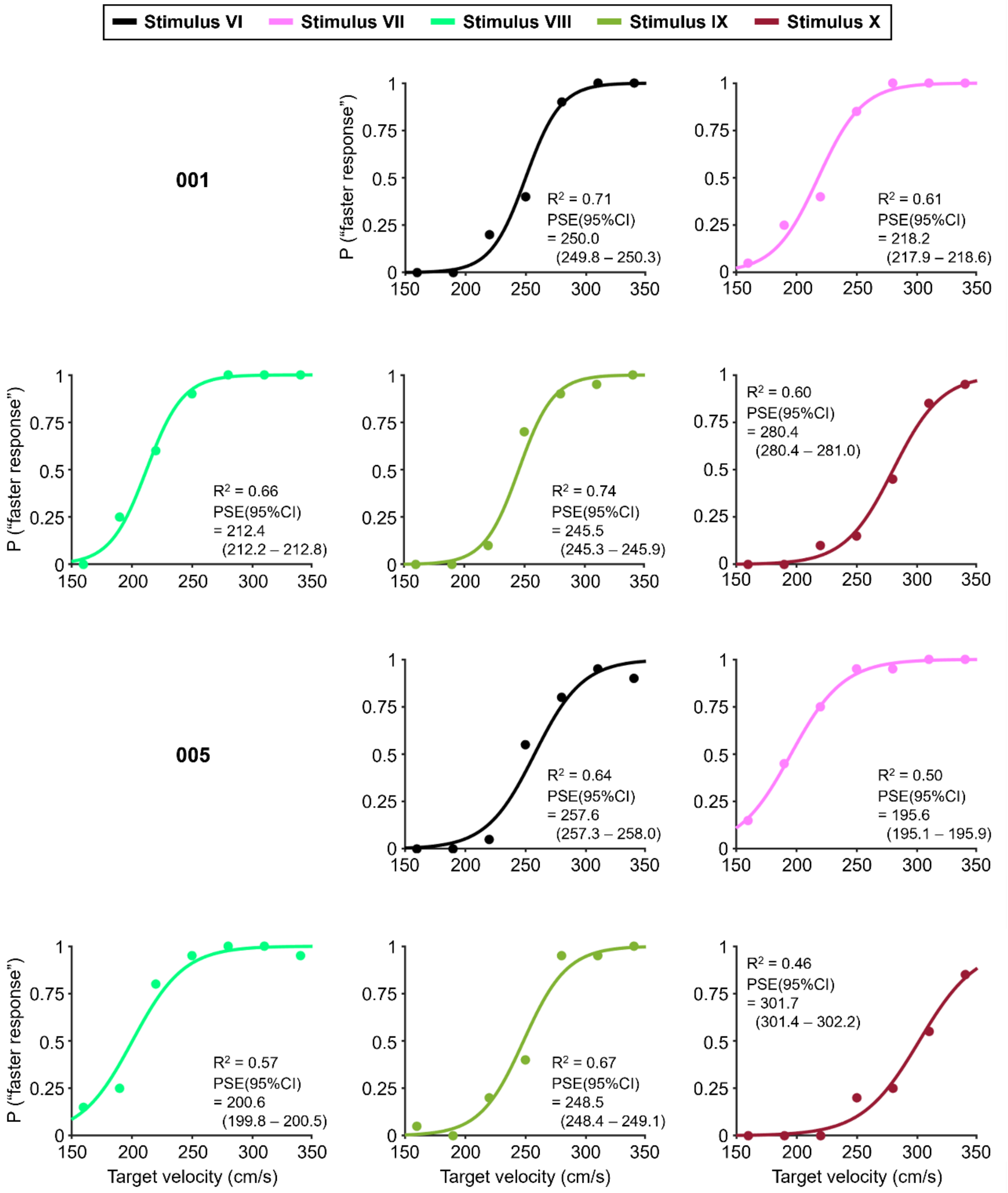
Stimulus VI
Stimulus VII
Stimulus VIII
Stimulus IX
Stimulus X
001
005
P ("faster response")
Target velocity (cm/s)
R² = 0.71 PSE(95%CI) = 250.0 (249.8 – 250.3)
R² = 0.61 PSE(95%CI) = 218.2 (217.9 – 218.6)
R² = 0.66 PSE(95%CI) = 212.4 (212.2 – 212.8)
R² = 0.74 PSE(95%CI) = 245.5 (245.3 – 245.9)
R² = 0.60 PSE(95%CI) = 280.4 (280.4 – 281.0)
R² = 0.64 PSE(95%CI) = 257.6 (257.3 – 258.0)
R² = 0.50 PSE(95%CI) = 195.6 (195.1 – 195.9)
R² = 0.57 PSE(95%CI) = 200.6 (199.8 – 200.5)
R² = 0.67 PSE(95%CI) = 248.5 (248.4 – 249.1)
R² = 0.46 PSE(95%CI) = 301.7 (301.4 – 302.2)

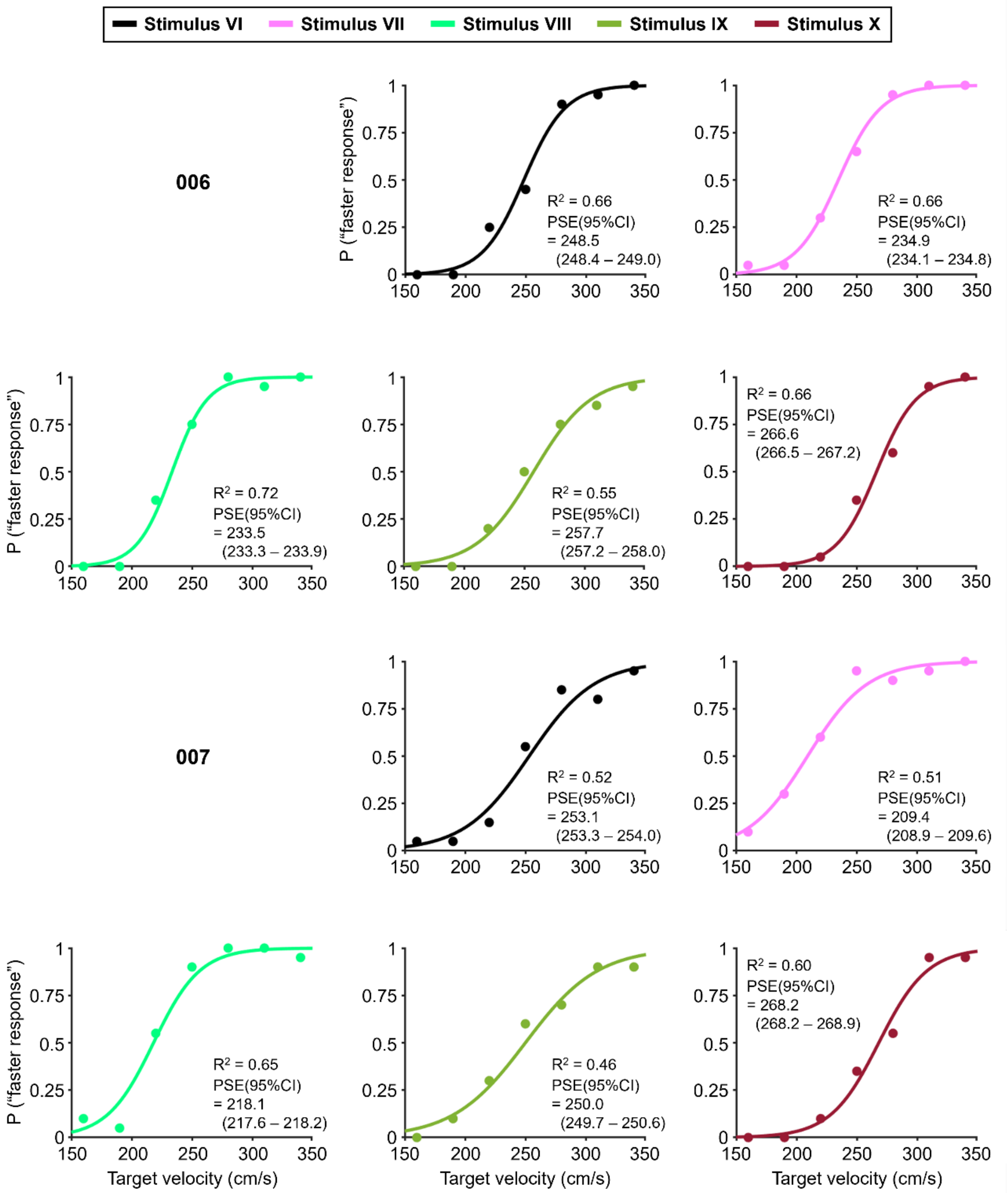

Stimulus VI
Stimulus VII
Stimulus VIII
Stimulus IX
Stimulus X
006
007
P (“faster response”)
Target velocity (cm/s)
R² = 0.66 PSE(95%CI) = 248.5 (248.4 – 249.0)
R² = 0.66 PSE(95%CI) = 234.9 (234.1 – 234.8)
R² = 0.72 PSE(95%CI) = 233.5 (233.3 – 233.9)
R² = 0.55 PSE(95%CI) = 257.7 (257.2 – 258.0)
R² = 0.66 PSE(95%CI) = 266.6 (266.5 – 267.2)
R² = 0.52 PSE(95%CI) = 253.1 (253.3 – 254.0)
R² = 0.51 PSE(95%CI) = 209.4 (208.9 – 209.6)
R² = 0.65 PSE(95%CI) = 218.1 (217.6 – 218.2)
R² = 0.46 PSE(95%CI) = 250.0 (249.7 – 250.6)
R² = 0.60 PSE(95%CI) = 268.2 (268.2 – 268.9)

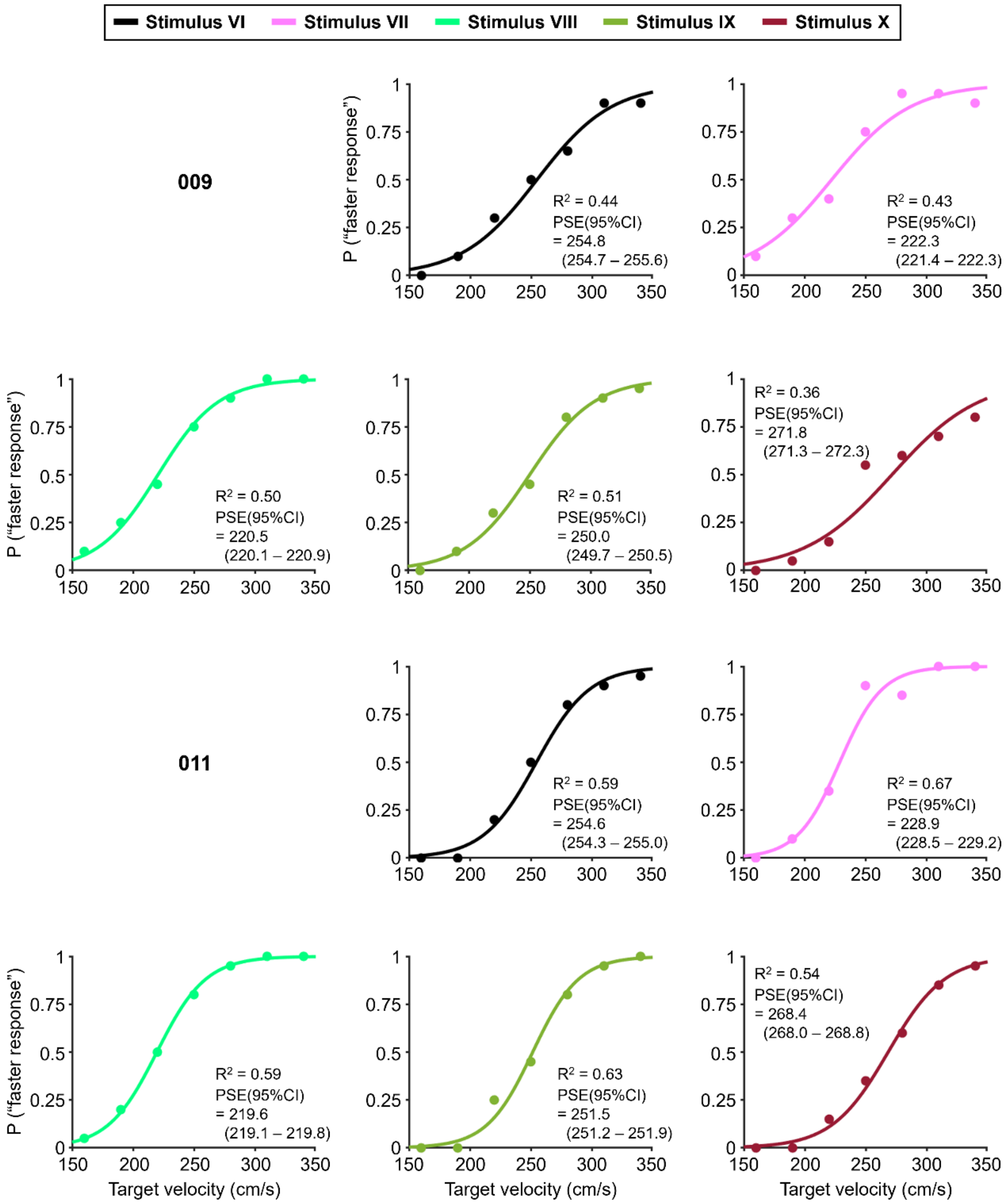

Stimulus VI
Stimulus VII
Stimulus VIII
Stimulus IX
Stimulus X
009
011
P ("faster response")
Target velocity (cm/s)
R² = 0.44 PSE(95%CI) = 254.8 (254.7 – 255.6)
R² = 0.43 PSE(95%CI) = 222.3 (221.4 – 222.3)
R² = 0.50 PSE(95%CI) = 220.5 (220.1 – 220.9)
R² = 0.51 PSE(95%CI) = 250.0 (249.7 – 250.5)
R² = 0.36 PSE(95%CI) = 271.8 (271.3 – 272.3)
R² = 0.59 PSE(95%CI) = 254.6 (254.3 – 255.0)
R² = 0.67 PSE(95%CI) = 228.9 (228.5 – 229.2)
R² = 0.59 PSE(95%CI) = 219.6 (219.1 – 219.8)
R² = 0.63 PSE(95%CI) = 251.5 (251.2 – 251.9)
R² = 0.54 PSE(95%CI) = 268.4 (268.0 – 268.8)

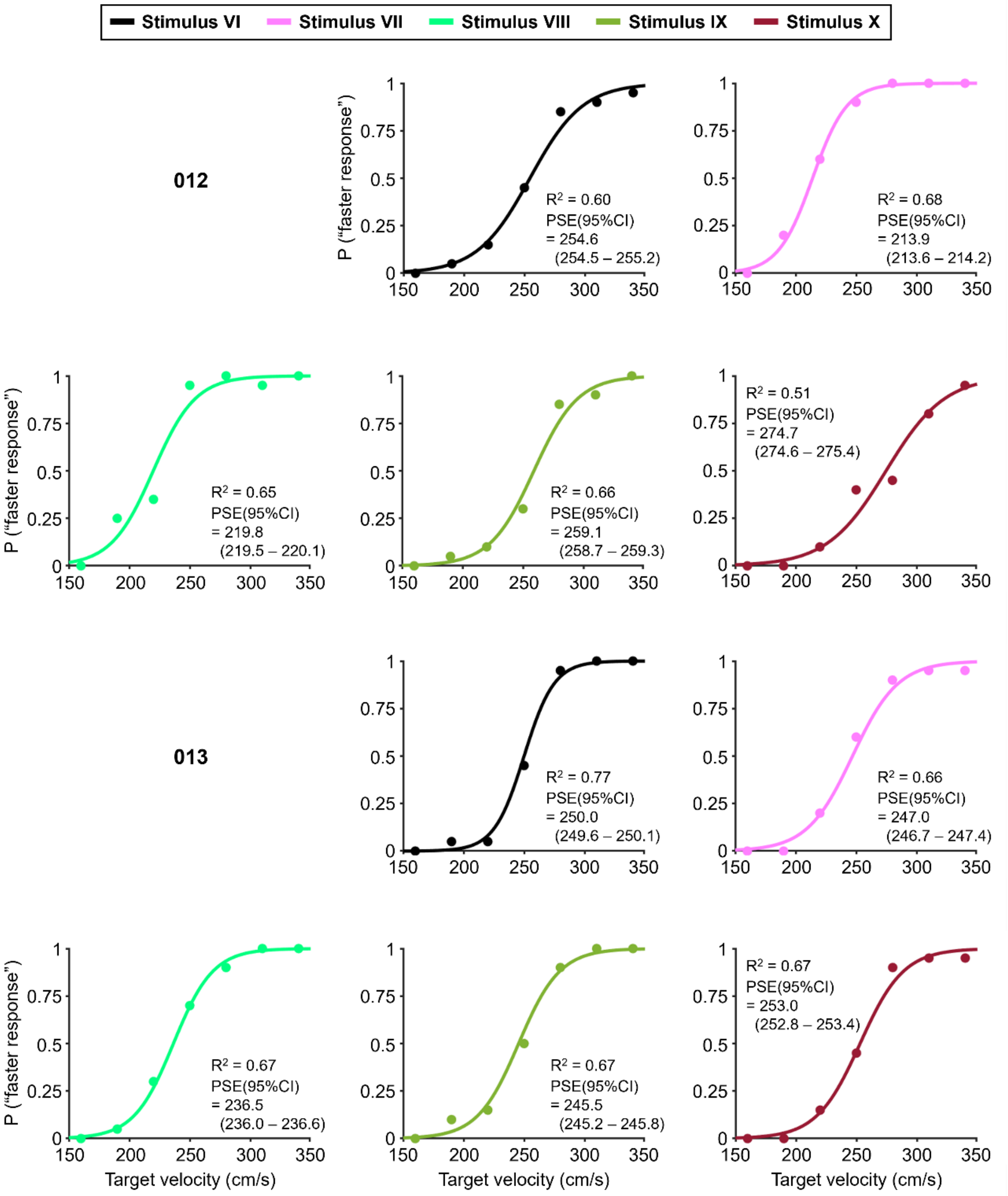
Stimulus VI
Stimulus VII
Stimulus VIII
Stimulus IX
Stimulus X
012
013
P (“faster response”)
Target velocity (cm/s)
R² = 0.60 PSE(95%CI) = 254.6 (254.5 – 255.2)
R² = 0.68 PSE(95%CI) = 213.9 (213.6 – 214.2)
R² = 0.65 PSE(95%CI) = 219.8 (219.5 – 220.1)
R² = 0.66 PSE(95%CI) = 259.1 (258.7 – 259.3)
R² = 0.51 PSE(95%CI) = 274.7 (274.6 – 275.4)
R² = 0.77 PSE(95%CI) = 250.0 (249.6 – 250.1)
R² = 0.66 PSE(95%CI) = 247.0 (246.7 – 247.4)
R² = 0.67 PSE(95%CI) = 236.5 (236.0 – 236.6)
R² = 0.67 PSE(95%CI) = 245.5 (245.2 – 245.8)
R² = 0.67 PSE(95%CI) = 253.0 (252.8 – 253.4)

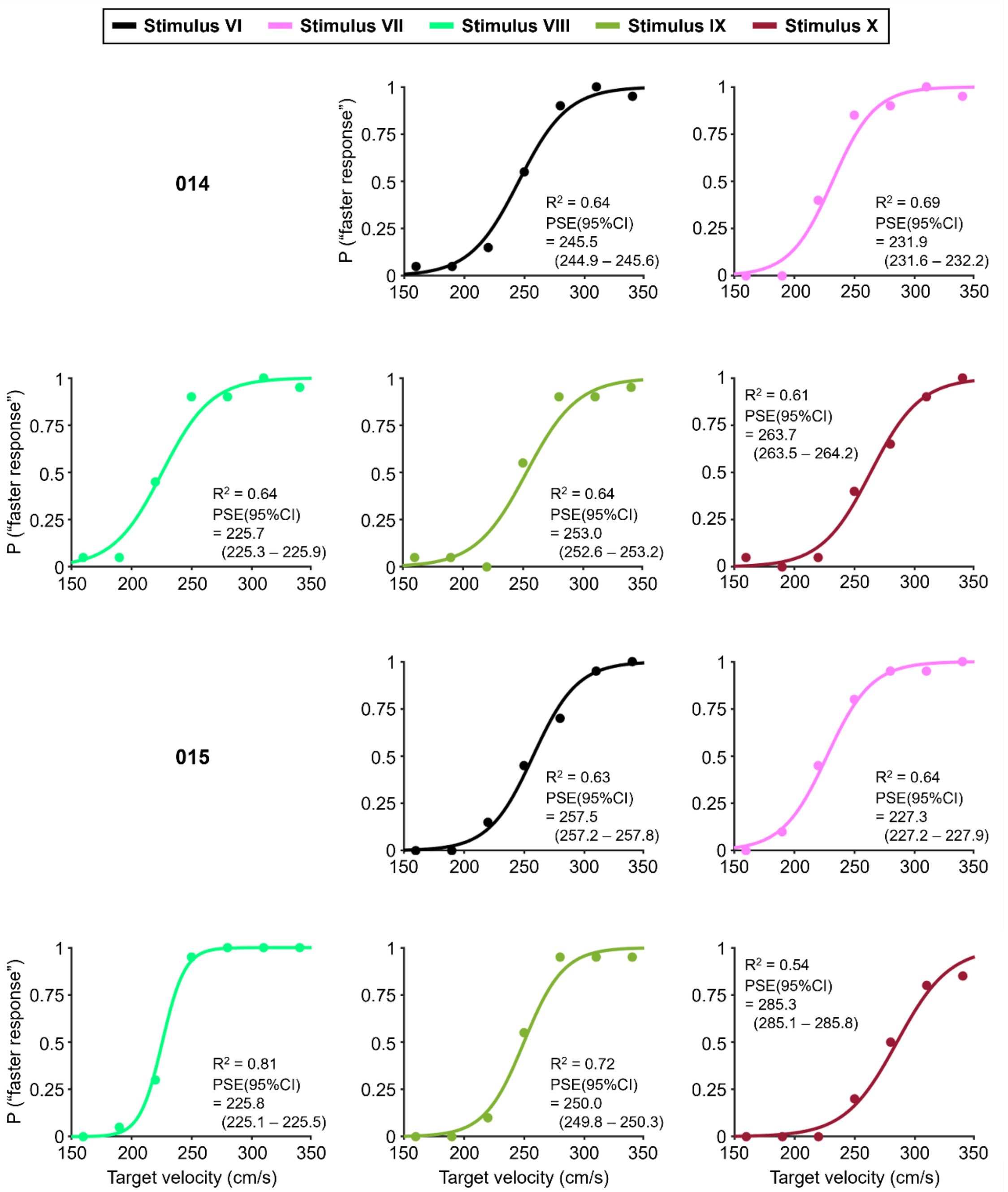

ESM 2 The psychometric functions in each stimulus for each participant in Experiment 2. The numbers (e.g. 001, 005) at the upper left part in each chart represent the participants. Each dot shows the probability that a participant indicated that the comparison stimulus for each target velocity was faster than the standard stimulus. The R^2 indicates the coefficient of determination of psychometric functions. The PSE(95%CI) represents the point of subjective equality based on the psychometric function (the lower bound - the upper bound in 95 % confidence interval of the PSE).
